# Supplementary material for: Some Novel Cobalt Diphenylphosphine Complexes: Synthesis, Characterization, and Behavior in the Polymerization of 1,3-Butadiene
Source: Molecules. 2021 Jul 2;26(13):4067. doi: 10.3390/molecules26134067 (PMC8271660; doi:10.3390/molecules26134067)
Supplement: Supplementary file 1 [file molecules-26-04067-s001.zip › molecules-1272889-supplementary.pdf]

molecules

MDPI

Supplementary Information

Some Novel Cobalt Diphenylphosphine Complexes: Synthesis, Characterization, and Behavior in the Polymerization of 1,3-Butadiene

Giovanni Ricci [1,*], Giuseppe Leone [1], Giorgia Zanchin [1], Benedetta Palucci [1], Alessandra Forni [1a], Anna Sommazzi [2], Francesco Masi [3], Stefano Zacchini [4], Massimo Guelfi [5] and Guido Pampaloni [5]

[1] CNR- Istituto di Scienze e Tecnologie Chimiche "Giulio Natta" (SCITEC), Via Corti 12, I-20133 Milano, Italy; giuseppe.leone@scitec.cnr.it (G.L.); giorgia.zanchin@scitec.cnr.it (G.Z.); benedetta.palucci@scitec.cnr.it (B.P.)
[1a] CNR- Istituto di Scienze e Tecnologie Chimiche "Giulio Natta" (SCITEC), Via Golgi 19, I-20133 Milano, Italy; alessandra.forni@scitec.cnr.it
[2] Versalis S.p.A.–Centro Ricerca Novara, Via Fauser 4, I-28100 Novara, Italy; anna.sommazzi@versalis.eni.com
[3] Versalis S.p.A.-R&D Partner Catalysis, Piazza Boldrini 1, I-20097 San Donato Milanese (MI), Italy; francesco.masi@versalis.eni.com
[4] Dipartimento di Chimica Industriale ''Toso Montanari'', Università di Bologna, Viale Risorgimento 4, I-40136 Bologna, Italy; stefano.zacchini@unibo.it
[5] Dipartimento di Chimica e Chimica Industriale, Università di Pisa, Via Moruzzi 13, I-56124 Pisa, Italy; massimo.guelfi@unipi.it (M.G.); guido.pampaloni@unipi.it (G.P.)
* Correspondence: giovanni.ricci@scitec.cnr.it

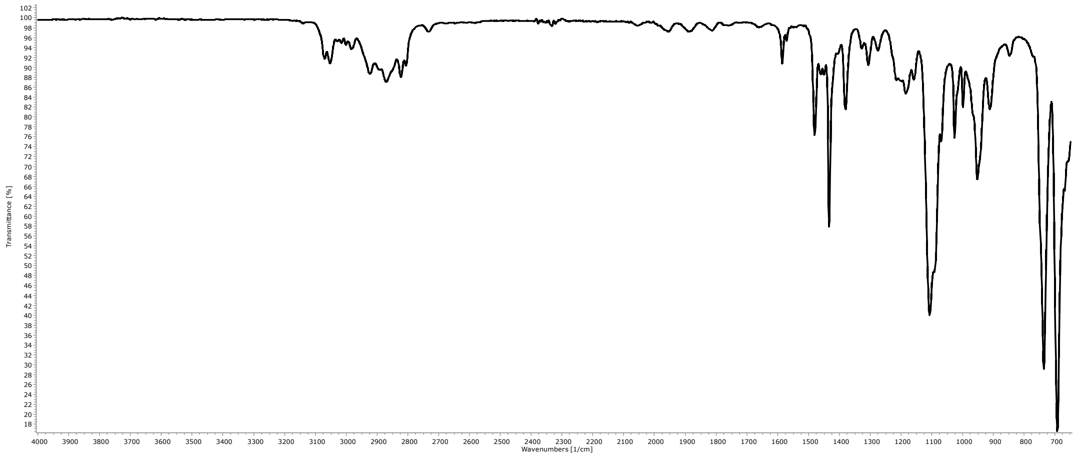

Figure S1. FT-IR spectrum of (2-methoxyethyl)diphenylphosphine.

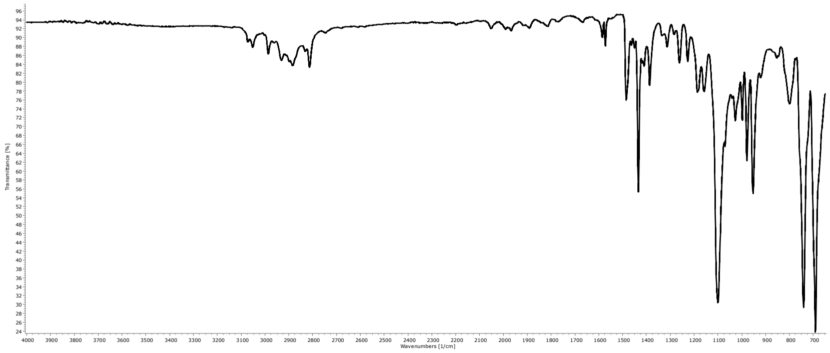

Figure S1A. FT-IR spectrum of [(2-methoxyethyl)diphenylphosphine]2 cobalt dichloride (1).

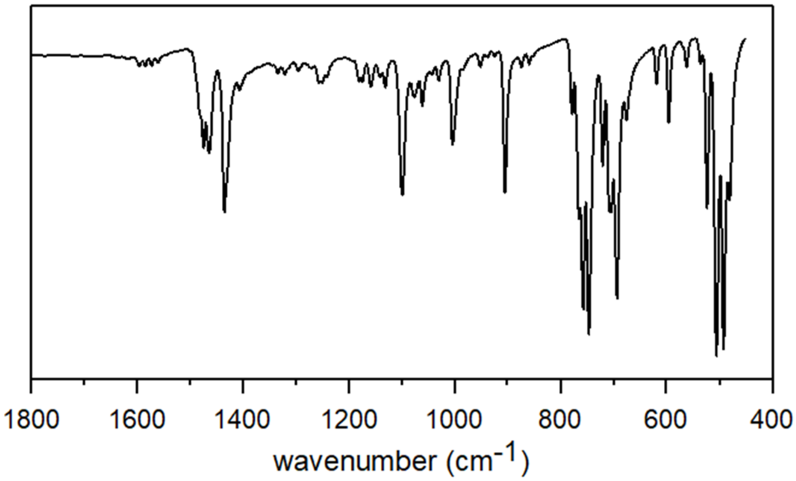

Figure S2. FT-IR spectrum of [(2-methoxyphenyl)diphenylphosphine]2 cobalt dichloride (2).

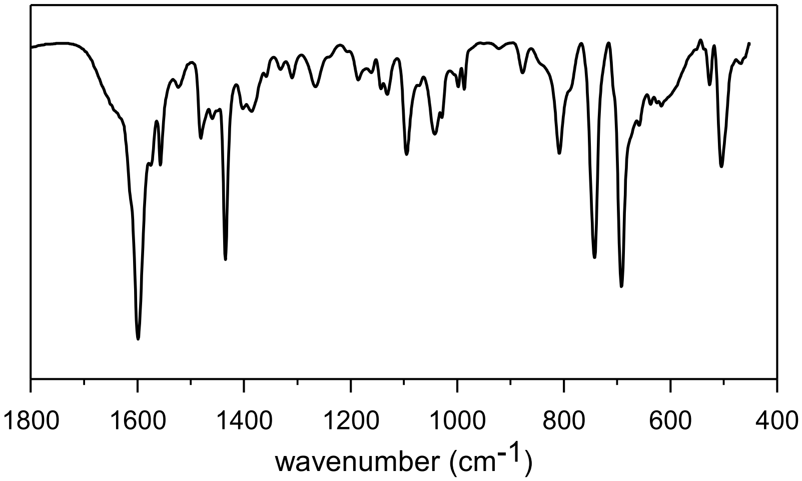

Figure S3. FT-IR spectrum of [2-(1,1-dimethylpropyl)-6-(diphenylphosphino)pyridine]2 cobalt dichloride **(3)**.

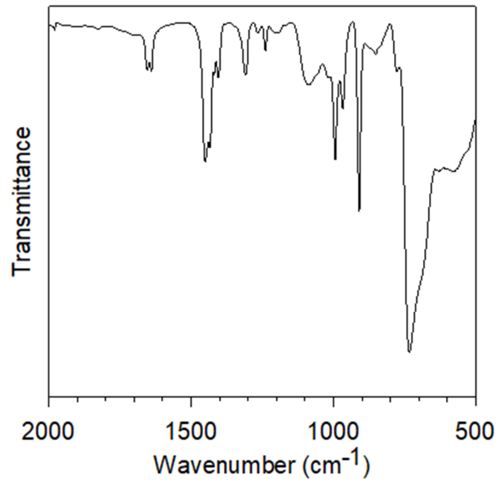

Figure S4. FT-IR spectrum of the polybutadiene of Table 3, run 1.

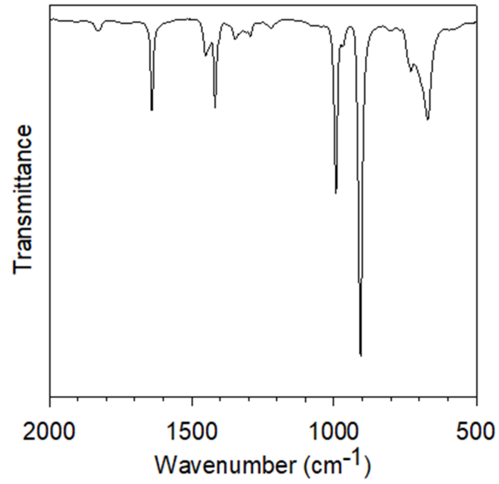

Figure S5. FT-IR spectrum of the polybutadiene of Table 3, run 2.

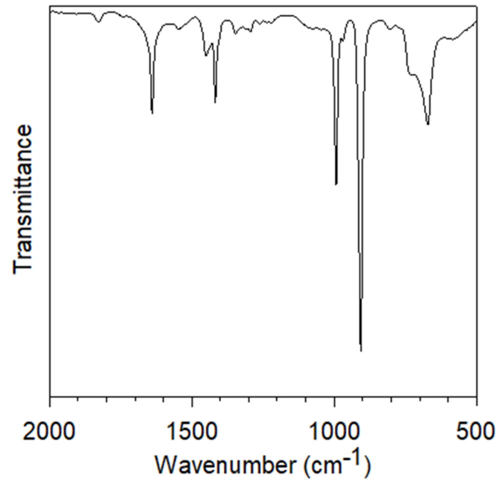

Figure S6. FT-IR spectrum of the polybutadiene of Table 3, run 3.

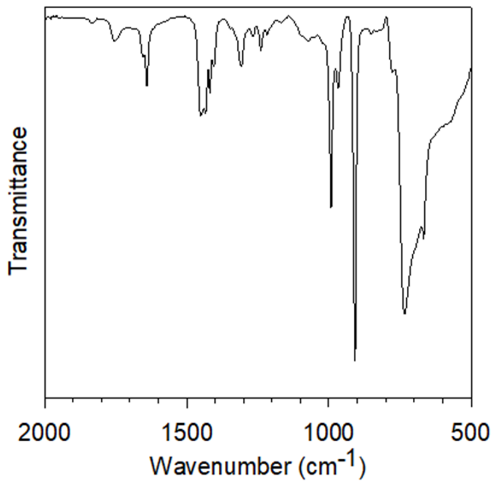

Figure S7. FT-IR spectrum of the polybutadiene of Table 3, run 4.

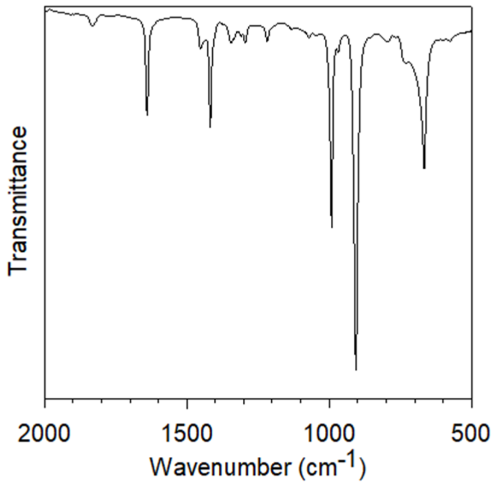

Figure S8. FT-IR spectrum of the polybutadiene of Table 3, run 5.

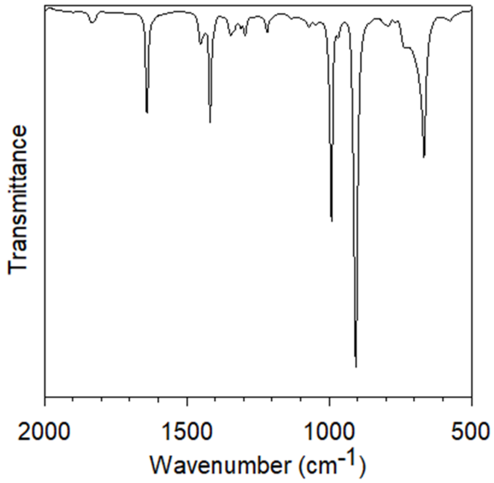

Figure S9. FT-IR spectrum of the polybutadiene of Table 3, run 6.

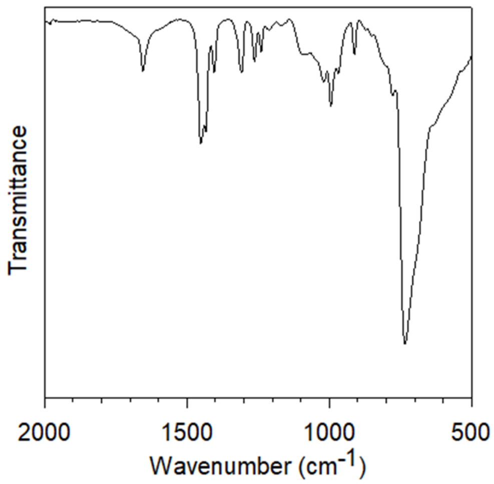

Figure S10. FT-IR spectrum of the polybutadiene of Table 3, run 7.

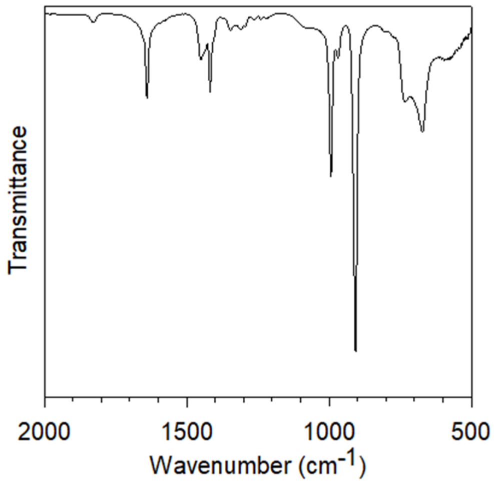

Figure S11. FT-IR spectrum of the polybutadiene of Table 3, run 8.

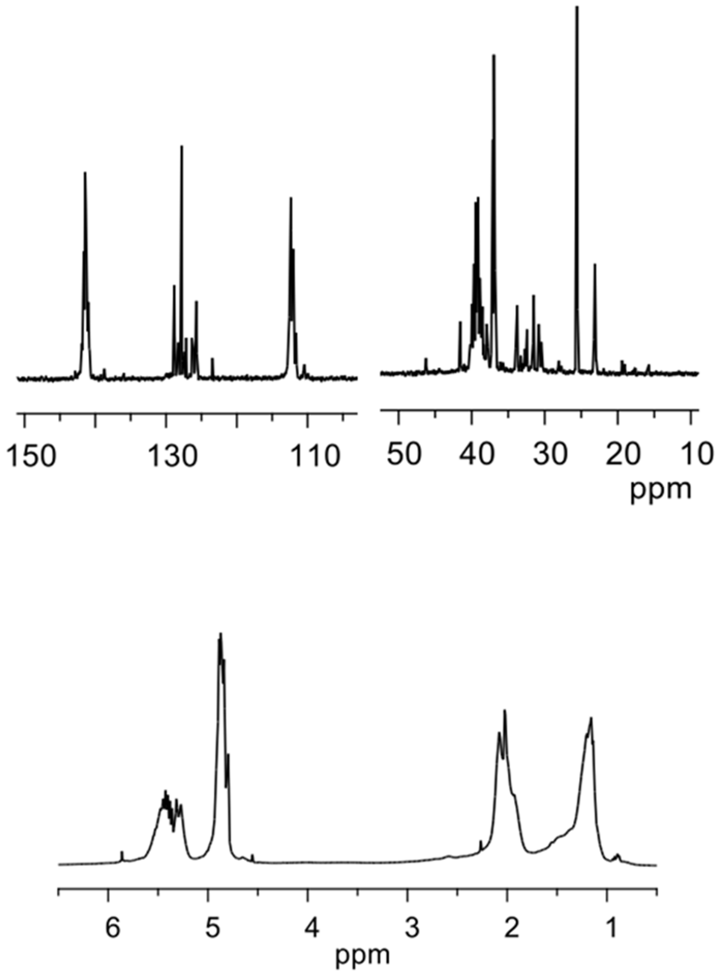

Figure S12. NMR spectra (^{1}H down, ^{13}C up) of the polybutadiene of Table 3, run 2.

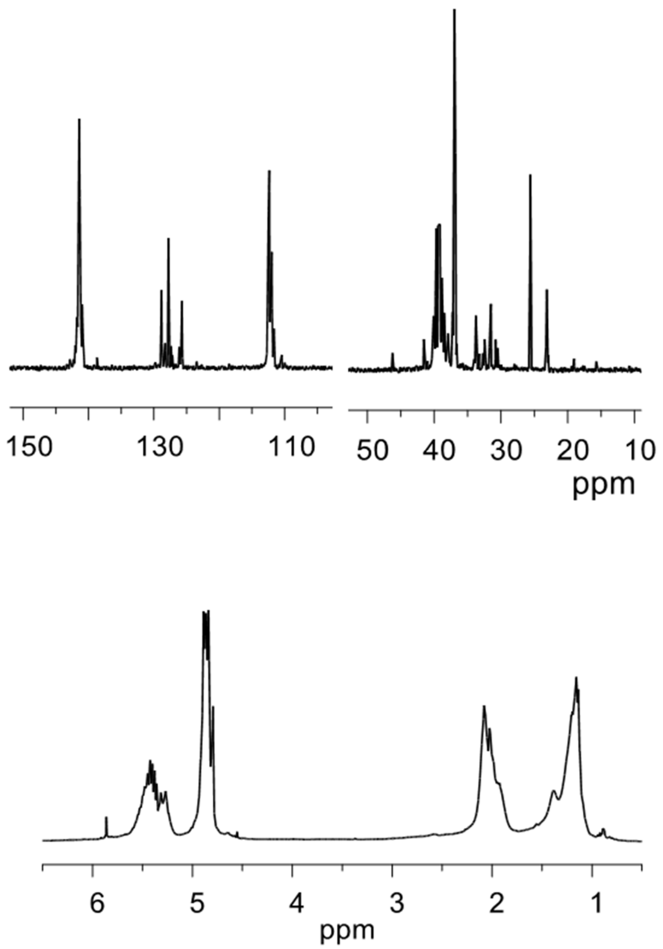

Figure S13. NMR spectra (^{1}H down, ^{13}C up) of the polybutadiene of Table 3, run 3.

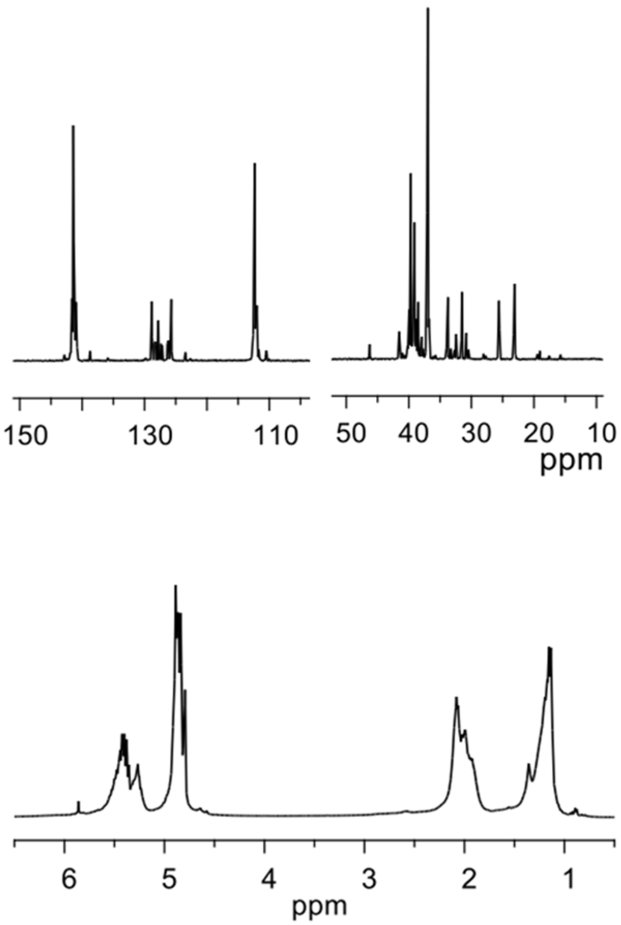

Figure S14. NMR spectra (^{1}H down, ^{13}C up) of the polybutadiene of Table 3, run 5.

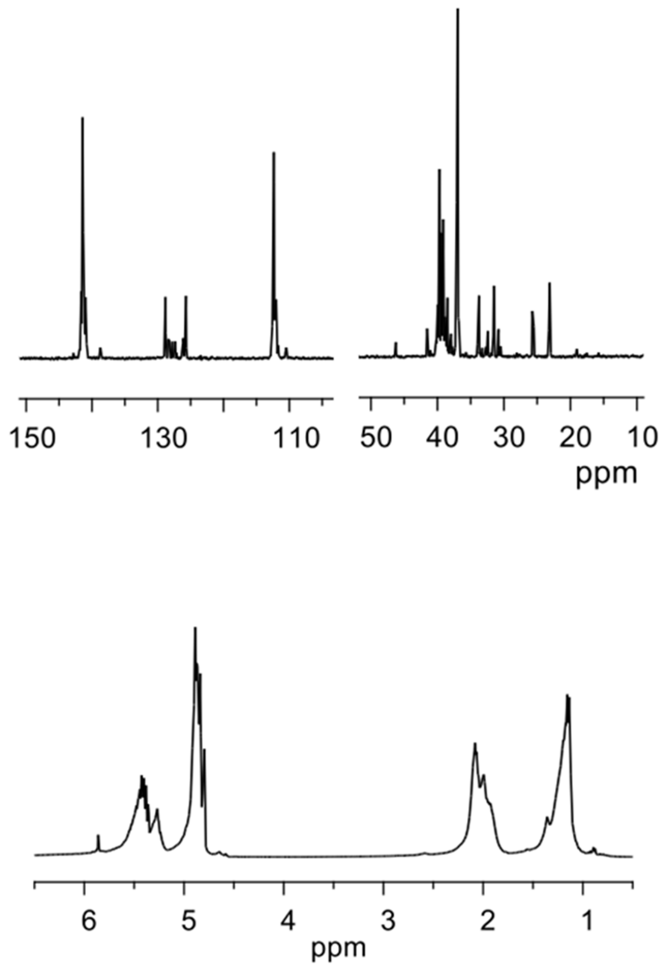

Figure S15. NMR spectra (^{1}H down, ^{13}C up) of the polybutadiene of Table 3, run 6.

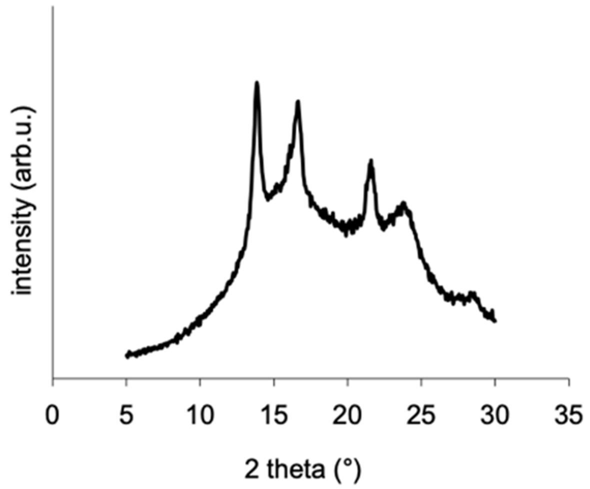

Figure S16. XRD spectrum of the polybutadiene of Table 3, run 5.

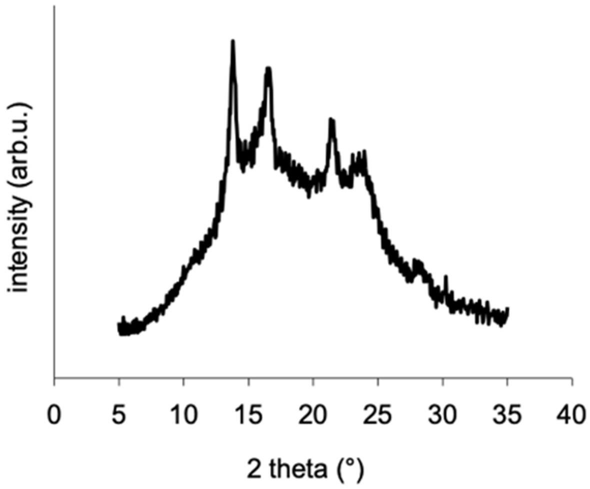

Figure S17. XRD spectrum of the polybutadiene of Table 3, run 6.
